# Spatial Spillover Effects of Directed Technical Change on Urban Carbon Intensity, Based on 283 Cities in China from 2008 to 2019

**DOI:** 10.3390/ijerph19031679

**Published:** 2022-02-01

**Authors:** Hui Zhang, Haiqian Ke

**Affiliations:** 1 Fanli Business School, Nanyang Institute of Technology, Nanyang 473000, China; zhanghuinylg@163.com; 2 Institute of Central China Development, Wuhan University, Wuhan 430072, China

**Keywords:** directed technical change, spatial durbin model, carbon intensity, urban sustainable development

## Abstract

Technical change essentially drives regional social and economic development, and how technical change influences the regional sustainable development of the ecological environment is also of concern. However, technical change is not always neutral, so how does directed technical change affect urban carbon intensity? Is there a spatial spillover effect between these two? In order to answer these above questions, this article first explores the relationship between directed technical change and carbon intensity through the spatial Durbin model; then, it separately analyses whether the relationship between the two in low-carbon and non-low-carbon cities will differ; finally, we performed a robustness test by replacing weights, replacing the explained variable with a lag of one period, and replacing the explained variable. The conclusions are as follows: (1) There is a positive spatial correlation between the carbon intensity of Chinese cities—that is, there is a positive interaction between the carbon intensity of local cities and of neighboring cities. For every 1% change in the carbon intensity of neighboring cities, the carbon intensity of local cities changes by 0.1027% in the same direction. (2) The directed technical change has a significant inhibitory effect on urban carbon intensity, whether in local cities or neighboring cities. However, it is worth mentioning that the direct negative effect is greater in local cities than in neighboring cities. (3) The directed technical change in low-carbon cities has a stronger inhibitory effect on carbon intensity, with a direct effect coefficient of −0.5346 and an indirect effect coefficient of −0.2616. Due to less green policy support in non-low-carbon cities, the inhibitory effect of directed technical change on carbon intensity is weakened; even if the direct effects and indirect effects are superimposed, it is only −0.0510 rather than −0.7962 for low-carbon cities.

## 1. Introduction

Climate change caused by the greenhouse effect is one of the greatest threats to human life on a global scale, because it endangers the ecological security of the Earth and, thus, the survival and development of human beings [1]. For example, a one meter rise in sea levels caused by the melting of glaciers would directly affect around one billion people, and also approximately one-third of the world’s arable land [2]. Hence, the IPCC and the Kyoto Protocol have jointly promoted the process of global cooperation in addressing climate change, and established the goal of preventing the increase in the surface temperature from exceeding 2 degrees Celsius in the future. The core countermeasure is to control and to reduce greenhouse gas emissions globally. In particular, there is an urgent need to control CO_2_ emissions and the growth rate of fossil energy consumption [2].

In the past 100 years, developed countries have undergone industrialization successively, consuming a large amount of the Earth’s natural resources—especially energy resources. However, some developing countries are currently entering the stage of industrialization, and increased energy consumption is an objective necessity for their economic and social development. Given the speed and scale of China’s economic development in recent years, China’s total carbon emissions have ranked first in the world since 2005 [3]. Therefore, China should give consideration to how to reduce the total amount of carbon emissions, and make continuous efforts in improving carbon productivity and reducing carbon intensity.

As the most direct and powerful means to reduce regional carbon emissions and the greenhouse effect, energy saving and emissions reduction are the inherent requirements for China to realize its own sustainable development, but also make important contributions to the global mitigation of climate change. The year 2006 was the year that China’s energy conservation and emissions reduction policy framework was formed. As China surpassed the US in total energy consumption for the first time in 2009, becoming the world’s largest energy consumer [4], China proposed to reduce energy consumption per unit of GDP by ~20% and to reduce the total amount of major pollutants by 10% during the 11th 5-year plan period, consistent with the goals proposed by 2020 to achieve carbon peak by 2030 and carbon neutrality by 2060 [5,6]. From the perspective of international experience, European developed countries have spent 55–60 years on reducing emissions, reaching the peak of carbon dioxide emissions in 1990–1996, and aiming to achieve zero carbon emissions by 2050. China’s goal is to achieve carbon peak and carbon neutrality in only 30 years; hence, there is no doubt that China’s process of reducing carbon emissions is full of hardships and challenges.

Green policy support and technical change are both important driving forces in reducing carbon emissions. On the one hand, most countries agree that optimizing the energy mix, market regulation, and political regulations are effective measures to reduce greenhouse gas emissions [7]. Additionally, saving non-renewable energy, developing renewable green energy, optimizing the energy structure, and reducing the release of gases harmful to the environment are sufficient and necessary conditions for achieving the targeted reductions in emissions. Additionally, in the process of achieving the above measures, technical change and support are also inseparable—the Porter hypothesis is the most powerful proof of this [8]. On the other hand, the US, Japan, and the European Union have all invested heavily into scientific research and the development of energy-saving and emissions-reducing technologies, and they have also achieved better environmental performance [9]. This confirms that technical change is an important driving force for regional energy conservation and global emissions reduction. However, from the perspective of endogenous economic growth theory, technical change is not always neutral—that is, the effect of technical change on productivity or the accumulation of production factors is not always similar. This may lead to an -unequal increase in the marginal output of factors such as capital and labor [10] which, in turn, can have varying degrees of impact on resource consumption, economic growth, and environmental quality. Simultaneously, as China is in a period of tightening resource and environmental constraints, promoting high-quality economic development, improving the environmental governance system, and committing to achieving green development, it is thus necessary to explore the impact of directed technical change on China’s total carbon emissions, as well as how to strengthen or mitigate said impact. Such exploration is thus conducive to an in-depth understanding of how to promote the transformation of China’s economic development to a green, low-carbon, and environmentally friendly mode.

## 2. Literature Review and Theoretical Hypotheses

Through reviewing the relevant literature, reducing carbon emissions mainly depends on the influence of factors such as energy prices, environmental policies, and technical change [11]. First of all, because carbon emissions have certain externalities, they come from unconscious and uncompensated economic activities. Therefore, the government allows a “Pigovian tax” policy to ensure that greenhouse gas emissions are no longer free, forcing enterprises to use greener technologies or fuels, thereby alleviating the decline in environmental quality [12]. The Chinese government has tried to curb the rise in total carbon emissions by controlling the carbon pricing, the related tax price, the number of carbon transactions, and the price of carbon credits [13]. For instance, in 2012 and 2014, China launched a carbon trading pilot in seven provinces and cities to try to internalize the external costs of enterprises’ emissions by controlling carbon pricing, and then formed China’s largest carbon trading market in 2021 [14]. Therefore, the increasing energy price due to the carbon tax will induce the increasing R&D investment in low-carbon technology, which will benefit the development of greener technologies. Secondly, market-based and command-based policies can impose mandatory emissions reduction targets and standards requiring enterprises to achieve emission reduction targets, respectively. In February 2017, the National Development and Reform Commission announced the third batch of national low-carbon city pilots, for which a total of 45 cities were selected [15], trialing cities with low-carbon economic development, citizens with a low-carbon lifestyle, and a low-carbon society under government management. Therefore, theoretically speaking, the support of low-carbon policies should make cities more successful in reducing carbon emissions. Third, technical change is a key factor in reducing emission reduction costs in the future, because more advanced technologies are often greener. For example, solar energy becomes cheaper faster than coal, so the costs of reducing carbon emissions are lower. The chemical industry, steel industry, construction industry, and other fields have achieved multiple milestones in energy saving, consumption reduction, and pollution reduction due to the embedded energy-saving technology, clean production technology, and the recycling of waste, showing the important role of technical change in China’s realization of carbon emission reductions. Specifically, technical change can be used to ease the contradiction between economic growth and the continuously increasing carbon emissions in the following four ways: First, technical changes can effectively reduce the dependence on and overuse of energy, simultaneously expanding green energy use without significantly slowing economic growth [16,17]. For example, the improvement of end-treatment technology and the establishment of a whole-process pollution reduction system can achieve carbon reduction [18]. Second, most scholars generally accept the view that the improvement of energy efficiency means that the same amount of energy input can bring more energy output [19]. Although some scholars have questioned whether higher energy efficiency means more energy consumption [20], it is undeniable that improved energy efficiency can also reduce energy costs. Third, as a terminal solution, carbon capture and storage can decompose the captured and stored carbon dioxide before, during, or after combustion, and transport and store it to a safe place [21], and the realization of all of these effective mitigation measures is inseparable from technical change and support. Fourth, with the advent of the era of big data, cloud technology has enabled the promotion and application of clean technology to be realized on a large scale at a faster speed or lower cost [22]. Therefore, the promotion of green policy support and technical change can help to reduce carbon emissions.

However, technical change is not always neutral. It has been found that technical change may be directed by factors such as factor price changes, R&D activities, trade, and FDI (foreign direct investment) technology spillovers. The idea of directed technical change was first proposed by Hicks (1932) [23], and it has been developed more comprehensively by Kennedy (1964), Samuelson (1965), and Drandakis and Phelps (1966) [24,25,26]. However, in recent theoretical research, the more commonly accepted theories mainly come from Acemoglu (1998; 2003; 2006; 2007) and Jones (2005) [27,28,29,30,31]. First of all, Acemoglu’s (2002) model is driven by the profits of “technology monopolists” rather than being induced by factor prices, so it can effectively distinguish between directed technical change and factor substitution effects [32]. At the same time, on the basis of the CES (constant elasticity of substitution) production function, Acemoglu proposed that technical progress changes the relative marginal productivity of factors in different proportions, so as to have different characteristics of saving or using factors. That is, if technical change increases the marginal productivity of factor *i* relative to factor *j*, then technical change is directed towards factor *i*; if the effect of directed technical change reduces the relative use of factor *i*, technical change is called “factor *i*-saving technical change”. Later, Jones (2005) argued that the shape of the production function and the direction of technical change were determined by the distribution of innovation [31]. Jones also explored the determinants of directed technical change under the Cobb–Douglas production function [31]. Despite Jones starting from a different perspective, his conclusion was in line with Acemoglu’s theory—both of them emphasized the reasonable micro-basis mechanism of directed technical change, and they have made some progress in theoretical models and empirical methods to promote the study area of directed technical change.

More importantly, many papers have confirmed that the energy efficiency of similar activities in different enterprises, fields, and countries is quite different, and the accelerated diffusion of energy technology is helpful in achieving energy conservation and emissions reduction [33,34,35,36,37]. This means that the emissions reduction effects brought by technical change have a certain degree of spatial relevance and spatial difference. In 2010, Sun calculated the average annual energy-saving rate of each province in China from 2006 to 2010, and found that the average annual energy-saving rate of neighboring cities has a certain degree of similarity [38]; this reflects how China’s energy-saving rate has a certain geographical correlation. Additionally, the similarity within the four plates and the difference between the four plates in China are also significant reasons for the spatial correlation of regional carbon emissions. Yao and Yu (2011) used the DEA method to reveal that the carbon intensity in the eastern and central provinces was significantly inhibited by technical change, while the carbon intensity in the northeast and western regions was increased by technical change [39]. Based on China’s provincial-level data, Wei and Yang (2010) found that there were regional differences in the effects of technology on emissions reduction [40]. In view of this, why does the influence of technical change on carbon emissions have regional agglomeration and differences, and can spatial correlation and difference strengthen or alleviate the influence of directed technical change on carbon emissions? This series of questions are worthy of further in-depth investigation.

Therefore, on the basis of the spatial model, this article will first explore whether there is an urban-directed technical change in China, and attempt to ascertain the effect of directed technical change on urban carbon emissions. In this article, the investment-biased technology change technique (IBTECH) proposed by Fare et al. (1994; 1997) is used to measure the directed technical change of 283 cities in China [41,42,43,44,45]. This method is based on the non-parametric data envelopment analysis (DEA) method, and is further decomposed on the basis of the Malmquist total factor productivity index. The advantage of this method is that it is not restricted by the shape of the production function or factor prices. In addition, carbon intensity is the ratio of the total amount of regional carbon emissions to the total regional GDP; this is mainly because the concept of carbon intensity is more objective than the total amount of carbon emissions itself, because the influence of factors such as population size and area size on the measurement results can be removed. At the same time, carbon intensity reflects the output efficiency of carbon emissions. As per capita GDP continues to increase, carbon intensity is expected to decline. Therefore, carbon intensity can also reflect the balance between economic development and carbon emissions more comprehensively.

**Figure 1:**
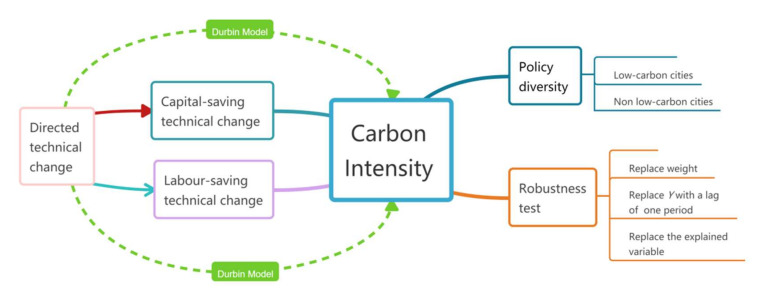
Roadmap of the paper.

Hence, this paper takes 283 prefecture-level cities in China as the research object in order to explore whether there is spatial correlation between urban carbon intensity and directed technical change, and then explores what kind of effect directed technical change will have on urban carbon intensity; the roadmap is reflected in Figure 1. The marginal contributions of this paper are as follows: Firstly, this paper innovatively expands and extends the scope of research on the interaction between directed technical change and urban carbon intensity from a spatial perspective. Secondly, this article examines whether and how official low-carbon policy support will affect the relationship between the two. For example, if directed technical change has a significant inhibitory effect on the local carbon intensity, it may nevertheless have a significant positive effect on the carbon intensity of neighboring cities. This result could be a strong reference for experts who study the spatial relationship between the two. At the same time, when the spatial correlation and mechanism of interaction between the two are more deeply understood, it may also help to explore the different underlying mechanisms of influence on different regions. Finally, this article verifies the relationship between the two from the city level, since cities are specific spatial carriers for achieving peak carbon and carbon neutrality goals, as they are more specific, objective, and realistic than national and provincial levels. Therefore, our findings may be helpful for relevant departments—such as the National Resources Commission of the State Council, the National Development and Reform Commission, the Ministry of Ecology and Environment, city councils, and relevant prefecture-level “carbon neutrality” monitoring and command platforms—helping them to form a better plan in the process of achieving China’s peak carbon and carbon neutrality. For example, if the above departments can recognize that the direction of technical change has a certain significant relationship with urban carbon intensity, through guiding or changing the direction of technical change, the inhibitory effect on urban carbon intensity can be enhanced or the enhancement effect on carbon intensity can be weakened.

## 3. Data and Models

### 3.1. Data

#### 3.1.1. Explained Variable: Carbon Intensity

This article adopts the reference method in the greenhouse gas emission inventory guidelines formulated by the IPCC in 2006 [46,47]—that is, to estimate energy consumption, unify the energy units, and then multiply the carbon content coefficient in order to calculate the total carbon emissions, and deduct the amount of carbon used as raw materials as well as the amount of carbon for non-energy use. Finally, the output is corrected with the oxidation coefficient and converted into carbon emissions. Afterwards, we measured China’s urban energy consumption from 2008 to 2019 by surveying the regional energy balance, and then calculated the carbon dioxide emissions from each city’s energy consumption, divided by the city’s gross domestic product, to determine the carbon intensity of one city:

(1) $$E_{co_{2}}=\frac{\sum_{i}A_{i}\cdot c_{i}}{GDP}$$

where *E_co_*2*__* is the regional total carbon intensity (tons/RMB 10,000), *A_i_* is the consumption of the *i*th energy (tons), and *c_i_* is the carbon dioxide emission coefficient of this energy.

(2) $$A_{i}=F_{i}+T_{i}+H_{i}+N_{i}$$

where *F_i_* is the terminal consumption of the ith energy of the region, *T_i_* is the consumption of the *i*th energy for power generation of the region, *H_i_* is the *i*th energy for heating consumption of the region, and *N_i_* is the *i*th consumption of industrial raw materials of the region.

**Figure 2:**
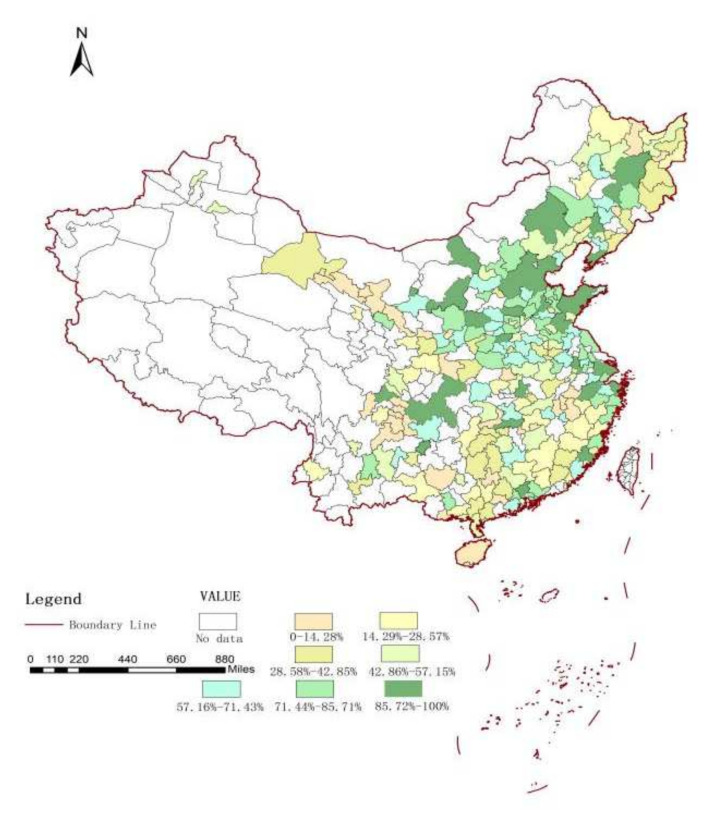
Carbon intensity in China, 2008.

**Figure 3:**
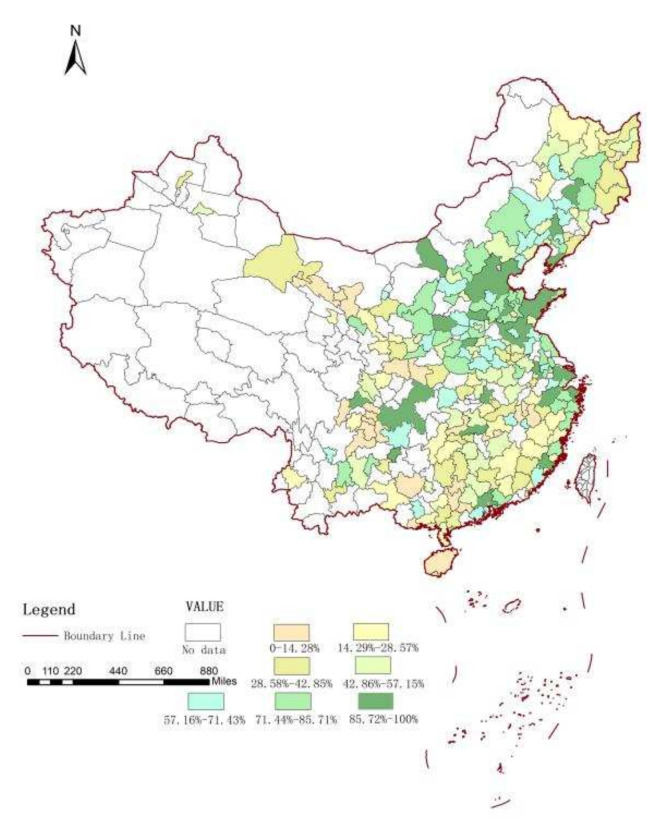
Carbon intensity in China, 2019.

Due to the scarcity of energy resources, the energy utilization between regions will flow and cross one another. Considering the spillover and diffusion effects of energy-saving technologies, energy consumption is influenced not only by local economic development strategies, technical change, and other factors, but also by these factors in surrounding areas. In addition, the frequent economic cooperation activities, energy-saving scientific and technical exchanges, and the radiation effects of energy-saving policies among Chinese cities have caused carbon emissions to be affected by local regions and adjacent regions; this is also the cause of the spatial concentration and correlation of urban carbon emissions in China. As we can see, this paper uses ArcGIS to draw the spatial distribution map of China’s urban carbon intensity from 2008 to 2019 in Figure 2 and Figure 3; we then divided all 283 cities into 7 groups (the urban carbon intensity of China is sorted into seven groups based on one-seventh of 100%. For example, group 1 represents 0–14.29% (1/7), and group 7 represents 85.72–100%). Greener color indicates higher carbon intensity, while yellower color indicates lower carbon intensity; the unit of urban carbon intensity is tons/RMB 10,000. It is natural to expect some correlation between the carbon intensity of Chinese cities. In fact, carbon intensity in 2008–2019 was on a downward trend, especially in the eastern region. However, we can also see that there was little change in carbon intensity in the central and western regions of China.

#### 3.1.2. Core Explanatory Variables: Directed Technical Change

Based on the use of DEA/Malmquist index to measure total factor productivity, Fare et al. (1997) decomposed the Malmquist index into the following parts: technical efficiency change index (FFCH) and technology change index (TECH) [41]; they also decomposed the technology change index into the technology scale change (MATECH), the output-biased technology change (OBTECH), and the input-biased technology change (IBTECH) indices. As IBTECH can effectively judge the direction of technical progress after combining the changes in the proportions of element input combinations between two adjacent periods, this article uses the IBTECH index to measure the directed technical progress [46,47,48], and the calculation method is as follows:

Let *x_t_* = (*x*_1*t*_,….., *x_Nt_*) be the vector of factor input in period *t*, and *yt* = (*y*_1*t*_,….., *y_Nt_*) be the vector of output in period *t*. The Shephard input distance function in period *t* can be defined as follows [47]:

(3) $$D_{t}{}^{i}(y,x)=\max\{\lambda :\frac{x}{\lambda }\in L^{t}(y)\}$$

(4) $$\mathrm{IBTECH}=\sqrt{\frac{D_{0}^{t+1}(y^{t+1},x^{t})}{D_{0}^{t}(y^{t+1},x^{t})}/\frac{D_{0}^{t+1}(y^{t},x^{t})}{D_{0}^{t}(y^{t},x^{t})}}$$

When *x*2_*t* + 1_/*x*1_*t* + 1_ < *x*2_*t*_/*x*1_*t*_ and *IBTECH* are both less than 1, the technical change is *x*1_-*saving*_, and the *IBTECH* becomes smaller the higher the degree of technical change directed towards *x*1_-*saving*_. When *x*2_*t* + 1_/*x*1_*t* + 1_ > *x*2_*t*_/*x*1_*t*_, the situation is the opposite. When *x*2_*t* + 1_/*x*1_*t* + 1_ = *x*2_*t*_/*x*1_*t*_, technical change is neutral. In this study, *x*1 is labor input, while *x*2 is capital input. In order to unify the two situations, this article sums up the opposite of the *IBTECH* value in *x*2_*t* + 1_/*x*1_*t* + 1_ > *x*2_*t*_/*x*1_*t*_, and *IBTECH* values under *x*2_*t* + 1_/*x*1_*t* + 1_ < *x*2_*t*_/*x*1_*t*_ remain unchanged so as to obtain the quantitative index of labor-saving technical change. Therefore, a large value of this index indicates that the directed technical change is more capital-saving [46,47]. This trend is also consistent with the findings of many scholars who investigated directed technical change in China [48]. Additionally, since the calculation process of directed technical change involves multiple indicators, different indicators often have different dimensions and units, which will affect the results of data analysis. To solve the comparability between data indicators, we used data normalization processing to convert the original data into decimals between (0, 1), after which each indicator was in the same order of magnitude, which is suitable for comprehensive comparative evaluation [49,50].

#### 3.1.3. Control Variables

Based on the STIRPAT model (Dietz and Rosa 1994) [51], in addition to directed technical change, population density, and average urban night lights, this paper selects the following five control variables (as shown in Table 1) from the economic, social, and environmental perspectives: the proportion of tertiary industry, foreign direct investment, total road passenger transport, the number of full-time college teachers, and the scale of pollution control investment [11,44,46,47]. Among them, population density, night light data, and total road passenger transport are expected to have the most positive effect on carbon intensity. However, the proportion of tertiary industry, foreign direct investment, the number of full-time college teachers, and the scale of pollution control investment are expected to have a suppressing effect on carbon intensity.

Table 1 provides a description of the explained variables, core explanatory variables, and control variables selected in this article, as well as the unit and the basic data source of each variable. Table 2 is the statistical description of main variables.

### 3.2. Models

#### 3.2.1. OLS Regression Model

In this study, the STIRPAT model (Dietz and Rosa, 1994) was used as the basic OLS regression model (ordinary least squares, which is the most fundamental form of regression analysis) to study the ecological environmental quality; the equation is expressed as follows [51]:

(5) $$\mathrm{IBTECH}=\sqrt{\frac{D_{0}^{t+1}(y^{t+1},x^{t})}{D_{0}^{t}(y^{t+1},x^{t})}/\frac{D_{0}^{t+1}(y^{t},x^{t})}{D_{0}^{t}(y^{t},x^{t})}}$$

where *I_it_* represents environmental impact, *P_it_* represents population size, A represents per capita wealth, *T_it_* refers to technology, and *e* is the error term. After obtaining the natural logarithms of both sides, more variables were added to the right side of the equation to enhance the richness of the model. Consequently, the benchmark model below was built to examine the relationship between urban carbon intensity and directed technical change:

(6) $${\mathrm{lncd}}_{\mathrm{it}}=\alpha_{0}+\alpha_{1}{\mathrm{lnpop}}_{\mathrm{it}}+\alpha_{2}{\mathrm{lnnl}}_{\mathrm{it}}+\alpha_{3}{\mathrm{lndtc}}_{\mathrm{it}}+\alpha_{4}X_{\mathrm{it}}+\varepsilon_{\mathrm{it}}$$

where *i* is the cross-sectional unit of the 283 Chinese cities investigated (due to the absence of necessary statistical data, 15 western cities among 298 prefecture-level cities were excluded herein), *t* is the year, population (*P_it_*) and technical level (*T_it_*) are represented by lnpopit and energy efficiency lndtcit, respectively, urban night light data (*lnnl_it_*) represent per capita wealth *A_it_*, and *lncd_it_* represents carbon intensity (the explained variable). *X_it_* is a group of control variables, which includes the proportion of tertiary industry, foreign direct investment, total road passenger transport, the number of full-time college teachers, and the scale of pollution control investment; *lndtc_it_* is the core explanatory variable, *ε* is a random disturbance term, and *α_0_*-*α_4_* are the estimated parameters.

#### 3.2.2. Spatial Regression Model

Because traditional measurement methods do not consider the spatial correlation between observations, there are certain limitations when studying multiple regional related issues. Scholars’ tentative research on spatial models mainly comes from SDM, SEM, and SDM, and there are also many academic papers on the impact of technical change on environmental quality [53,54,55]. However, based on the STIRPAT model, this paper takes the directed technical change as the core explanatory variable and carbon intensity as the explained variable—a method rarely seen in previous studies.

Spatial measurement models are generally divided into three types: spatial lag model (SLM), spatial error model (SEM), and spatial Durbin model (SDM) [56,57,58,59,60]. The SLM adds the spatial lag term of the explained variable to the general panel data model, indicating that the explanatory variable on a certain spatial unit is affected by the explanatory variable of the adjacent spatial unit. The SEM adds spatially related error terms—that is, the error term of a certain spatial unit model is considered to be affected by the adjacent spatial unit model error term. The SDM integrates the characteristics of the spatial lag model and the spatial error model. In all of the above models, the intensity of the influence of adjacent spatial units is represented by a spatial weight matrix. Therefore, this paper tries to propose an SLM model (Formula (7)), an SEM model (Formula (8)), and an SDM model (Formula (9)) to describe in depth the relationship between carbon intensity, population, economic development level, and directed technical change, as follows:

(7) $${\mathrm{lncd}}_{\mathrm{it}}=\delta \sum_{j=1}^{N}W_{\mathrm{ij}}{\mathrm{lncd}}_{\mathrm{jt}}+\phi +\alpha_{1}{\mathrm{lnpop}}_{\mathrm{it}}+\alpha_{2}{\mathrm{lnnl}}_{\mathrm{it}}+\alpha_{3}{\mathrm{lndtc}}_{\mathrm{it}}+c_{i}+\alpha_{t}+\varepsilon_{\mathrm{it}}$$

(8) $$\begin{array}{c} {\mathrm{lncd}}_{\mathrm{it}}=\phi +\alpha_{1}{\mathrm{lnpop}}_{\mathrm{it}}+\alpha_{2}{\mathrm{lnnl}}_{\mathrm{it}}+\alpha_{3}{\mathrm{lndtc}}_{\mathrm{it}}+c_{i}+\alpha_{t}+u_{\mathrm{it}}\\ u_{\mathrm{it}}=\rho \sum_{j=1}^{N}W_{\mathrm{ij}}u_{\mathrm{jt}}+\varepsilon_{\mathrm{it}} \end{array}$$

(9) $$\begin{array}{l} {\mathrm{lncd}}_{\mathrm{it}}=\delta \sum_{j=1}^{N}W_{\mathrm{ij}}{\mathrm{lncd}}_{\mathrm{jt}}+\phi +\alpha_{1}{\mathrm{lnpop}}_{\mathrm{it}}+\alpha_{2}{\mathrm{lnnl}}_{\mathrm{it}}+\alpha_{3}{\mathrm{lndtc}}_{\mathrm{it}}\\ +\sum_{j=1}^{N}W_{\mathrm{ij}}({\mathrm{lnpop}}_{\mathrm{it}}+{\mathrm{lnnl}}_{\mathrm{it}}+{\mathrm{lndtc}}_{\mathrm{it}})\theta +c_{i}+\alpha_{t}+\varepsilon_{\mathrm{it}} \end{array}$$

where *δ* is the spatial regression coefficient, which represents the influence of the explained variable *lncd_jt_* of the adjacent spatial unit on the explained variable *lncd_it_* of this spatial unit; if it is significantly positive, this means that the explained variable has obvious positive spatial overflow—that is, the increase in the variable of one spatial unit in the research scope corresponds to the increase in the variables of other spatial units; *u_it_* is the spatial autoregressive error term; *ρ* is the spatial error coefficient, which represents the influence of the adjacent spatial unit error term *u_it_* on the spatial unit error term *u_it_*; *θ* is the spatial lag term coefficient of the explanatory variable, which indicates the influence of the adjacent spatial unit explanatory variable *lndtc_it_* on the explanatory variable *lncd_it_* of this spatial unit; *N* is the number of spatial units, and ***W*** is the economic geographic spatial weight matrix. First, we used the LM test to determine whether the spatial lag effect and the spatial error effect were significant, and then used the Wald or *LR* tests to determine whether the spatial Durbin model can be simplified into a spatial lag model or a spatial error model. Assumption 1: *θ* = 0; Assumption 2: *θ* + *λβ* = 0. If Assumption 1 passes the significance test, the spatial Durbin model can be reduced to a spatial lag model, while if Assumption 2 passes the significance test, the spatial Durbin model can be reduced to a spatial error model [61,62].

#### 3.2.3. Direct and Indirect Effects Regression Model

From the mathematical structure of the SDM, it can be seen that the explanatory variable *y_local_* of a certain spatial unit is mainly affected by three aspects: the explained variable *y_neib_* of the adjacent space unit, the explanatory variable *x_local_* of this space unit, and the explanatory variable *x_neib_* of the adjacent space unit. In addition to directly affecting *y_local_*, the *x_local_* of this space unit can also affect the *y_neib_* of adjacent space units, and then transfer it to the space unit *y_local_* through the spatial autocorrelation of *y_neib_*. This comprehensive effect of *x_local_* on *y_local_* becomes a direct effect of explanatory variables; the *x_local_* of this space unit can directly affect the *y_neib_* of the adjacent space unit, or it can affect the *y_local_* of this space unit, and then transfer to the *y_neib_* of the adjacent space unit through the spatial autocorrelation of *y_local_*. This comprehensive effect of *x_local_* on *y_neib_* becomes an indirect effect, also known as the spatial spillover effect of explanatory variables. Therefore, the spatial Durbin model can be rewritten into the following form [61,62]:

(10) $$Y_{\mathrm{it}}={\left(1-\lambda W\right)}^{-1}\varphi_{N}+{\left(1-\lambda W\right)}^{-1}{\left(X_{t}\beta +WX_{t}\theta \right)}^{-1}+{\left(1-\lambda W\right)}^{-1}v_{t}^{*}$$

where *φ_N_* represents the space error term, meaning the error term with time and space effects. Therefore, the partial derivative of the explained variable *Y* with respect to the *k*th explanatory variable at a certain moment is:

(11) $$[\frac{\partial Y}{\partial X_{1k}}\cdot \frac{\partial Y}{\partial X_{Nk}}]=(I-\lambda W{)}^{-1} [\begin{array}{cccc} \beta_{k} & W_{12}\theta_{k} & \ldots & W_{1N}\theta_{k}\\ W_{21}\theta_{k} & \beta_{k} & \ldots & W_{2N}\theta_{k}\\ \ldots & \ldots & \ldots & \ldots \\ W_{N1}\theta_{k} & W_{N2}\theta_{k} & \ldots & \beta_{k} \end{array}]$$

where the mean of the diagonal elements on the right is the direct effect, and the mean of the sum of each row (column) of the off-diagonal elements is the indirect effect [63,64,65].

## 4. Results Analysis

### 4.1. OLS Regression Results

The results of Table 3 show the OLS regression results based on Formula (7). The capital-saving technical change suppress the increase in urban carbon intensity in China. One unit of capital-saving technical change could lead to a −0.0162 unit decrease in urban carbon intensity. This may be because highly skilled labor restrains the expansion of production by reducing capital inputs such as plants and machinery, and also reduces carbon intensity to some extent through the accumulation of human capital. Additionally, the population density, foreign direct investment, number of full-time college teachers, and pollution abatement show inhibitory effects on the urban carbon intensity of Chinese cities. However, the average night light data, tertiary industry proportion, and total road passenger transport all increase the urban carbon intensity significantly.

### 4.2. Spatial Regression Results

There are many methods of “spatial correlation or spatial structure test”; Moran’s I index is by far the earliest, most used, and most famous test in practice [66], and it is simpler in calculation than Wald and LR tests [67]. However, its disadvantage is that the test was not developed under the framework of the maximum likelihood method, and no alternative hypothesis is proposed [68]. Anselin found that the RS test has an asymptomatic standard when testing the spatial model of cross-sectional data, but the Wald and LR tests do not [69]. For the spatial error autocorrelation cross-sectional model with spatial lag-dependent variables, a robust LM test has further introduced joint, marginal, and conditional tests to verify the spatial model structure [56].

Therefore, as shown in Table 4, we carried out an LM test and a robust LM test for non-spatial interaction models with four forms: time and space are not fixed, space is fixed, time is fixed, and time and space are fixed; the results show that the spatial error effect cannot pass the 1% robustness test, except for the time–space-non-fixed effect, space-fixed effect, and time-fixed effect; the other kinds of spatial lag effect and spatial error effect pass the LM test and robust LM test, and the spatial lag effect is more significant than the spatial error effect, showing that the SDM cannot be reduced to the SLM or the SEM—that is, the SDM is optimal. Therefore, it is necessary to construct the SDM under the fixed form of space and time.

Table 5 shows the results based on the SDM. The coefficients and significance levels are overall improved compared to the OLS regression results from Table 3. Firstly, this shows that the spatial regression coefficient *δ* is significantly positive, indicating that the carbon intensity of Chinese cities is obviously affected by the carbon intensity of neighboring cities in the same direction, and also positively affects the carbon intensity of neighboring cities. This confirms that the carbon intensity of cities has obvious spatial spillovers—that is, for every 1% change in the carbon intensity of neighboring cities, the carbon intensity of the local city will change in the same direction by 0.1027%. Secondly, the significance test of the *t*-value of the regression coefficient *β* shows that the carbon intensity of Chinese cities is negatively affected by local capital-saving technical change, population density, foreign direct investment level, and pollution abatement; however, it is positively affected by night light data, the proportion of tertiary industries, and number of road passengers. Once again, the level of urban education shows no significant impact on carbon intensity. Finally, the *t*-value significance test of the spatial lag coefficient *θ* shows that the carbon intensity level of 283 cities in China is negatively affected by the capital-saving technical progress of neighboring cities. For neighboring cities, their population density, night light data, foreign direct investment level, proportion of tertiary industries, road passenger traffic, and pollution control investment all contribute to the improvement of the local city’s carbon intensity. In summary, the degree of carbon intensity in Chinese cities is affected by the local and neighboring regions’ capital-saving technical change, population density, night light data, foreign direct investment level, proportion of tertiary industries, number of road passengers, and pollution control investment. In addition, the capital-saving technical change, night light data, foreign investment level, and proportion of tertiary industries in neighboring cities play more important roles.

### 4.3. Results of Direct and Indirect Effects of Variables

Table 6 shows the further calculation of the direct and indirect effects of various influencing factors on urban carbon intensity. The *rho* value was 0.2857 and the significance test was passed at the level of 10%. This shows that urban carbon intensity still has a strong positive spatial correlation. The regression results first show that the direct and indirect effects of capital-saving technical are is significantly negative, and that the direct effect is greater than the indirect effect, indicating that the superposition of the direct effect and the indirect effect significantly inhibits the carbon intensity of the local city and its neighbors—that is, if the level of capital-saving technical change increases by 1 unit, the carbon intensity of the city and neighboring cities will decrease by 0.0472 and 0.0259 units, respectively, which means that the city’s overall carbon intensity will decrease by 0.0731 units in total. Secondly, the direct and indirect effects of average night light data, foreign investment level, the proportion of tertiary industries, and road passenger traffic are all in the same direction among local cities and neighboring cities; among them, night light data and road passenger traffic promote the carbon intensity of local cities and neighboring cities, meaning that the economic vitality of the region can drive the economic development of neighboring regions, which will inevitably use and consume more energy and increase the overall carbon intensity level. The increase in the level of local foreign investment is conducive to the imitation and learning of advanced knowledge and technology, and it can also play a demonstrable effect in neighboring areas. Because more advanced technology is often greener and more environmentally friendly, increasing the level of foreign investment can reduce the carbon intensity of local and neighboring cities; although a higher proportion of tertiary industry is accompanied by an increase in consumption, it is lower than the volume of resources consumed by the primary and secondary industries, so the overall increase in carbon emissions is significantly suppressed. The more the road passenger traffic, the wider the scope of social and economic activities between neighboring cities, and the higher the frequency of interaction between cities; therefore, road traffic plays an overall promoting role in carbon intensity. Third, the direct and indirect effects of population density, the number of full-time teachers in colleges and universities, and pollution control investment differ significantly or insignificantly. The increase in population density of local cities has a certain siphoning effect on the total population of neighboring cities. Therefore, the inhibitory effect of population agglomeration on carbon intensity mainly comes from the indirect effect of reducing the carbon intensity level of neighboring cities. The urban education level represented by the number of full-time university teachers does not show a significant correlation with carbon intensity. Meanwhile, a higher scale of pollution control investment has a significant direct inhibitory effect on the local city, and the coefficient is greater than its positive indirect effect. After the combined effect of the two, the overall carbon intensity of the city remains suppressed. In view of this, except for the educational level, all other factors have significant direct and indirect effects; among them, the spatial spillover effects of capital-saving technical change, population density, and the proportion of tertiary industries are the most obvious, significantly reducing the urban carbon intensity.

### 4.4. Robust Test

In order to separate the environmental policy from the regression results, as the former may influence the latter, 45 low-carbon cities and 238 non-low-carbon cities were selected for the regression of the SDM (in February 2017, the National Development and Reform Commission announced the list of the third batch of national low-carbon city pilots, and a total of 45 cities were selected). The results in Table 7 show that *rho* values of 0.1793 and 0.2667 both pass the significance test at the 5% level, indicating that there is still a strong positive spatial correlation of urban carbon intensity with directed technical change in China. Under the SDM, the capital-saving technical change of low-carbon cities has a stronger inhibitory effect on local carbon intensity, with a direct effect coefficient of −0.5346 and an indirect effect coefficient of −0.2616. This shows that the increase in capital-saving technical change can better improve resource utilization, increase the development of new energy technologies, and reduce pollutant emissions in the production process, thereby suppressing the carbon emissions of the local and neighboring areas. For non-low-carbon cities, due to the lack of support for green environmental protection policies, the inhibitory effect of capital-saving technical change on carbon intensity is weakened. Even if the direct effect and the indirect effect are superimposed together, the coefficient is only −0.0510, which is lower than the coefficient of −0.7962 for low-carbon cities. The analysis results of the remaining explanatory variables are generally consistent with the results of Table 3, except for the direct and indirect effects of population density on non-low-carbon cities, which are both shown to significantly promote carbon emissions. Most of the coefficients of the proportion of tertiary industries are significantly positive.

In Table 8, this paper presents three ways to further test the robustness of results: the replacement of geographic weights, the use of the dynamic SDM (the explanatory variable will lag one period), and the replacement of the explanatory variable as the carbon footprint. This shows that the capital-saving technical change still directly or indirectly shows a significant inhibitory effect on the urban carbon intensity in China, proving that the regression results in this article are robust. In addition, by comparing the magnitude of the coefficients, it can be seen that the capital-saving technical change still has a stronger inhibitory effect on the carbon intensity of the local area than that of the neighboring area—that is, the accelerated flow of capital resources in the region and its adjacent areas caused by the capital-saving technical change in the region can not only raise the level of environmental protection technology, but also play a role in reducing the carbon intensity; at the same time, due to the existence of the warning effect, it may also lead to an increasing demand for ecological environment improvement in the surrounding areas; therefore, the governments of the neighboring regions pay more attention to the degree of utilization of technical change, thereby restraining the carbon intensity of the neighboring regions. The performance of the remaining control variables is essentially the same as in Table 3.

## 5. Conclusions

Based on the SDM, this paper investigates the relationship between directed technical change (capital-saving) and carbon intensity, and then discusses whether the relationship will change in low-carbon cities and non-low-carbon cities. Finally, the robustness of the regression results in the case of replacement of weights, replacement of the *Y* lag period, and replacement of explained variables is explained. The conclusions are as follows: (1) The carbon intensity of Chinese cities is positively and significantly influenced between local cities and the neighboring cities; the carbon intensity of the local city will change by 0.1027% of a unit in the same direction as one unit of a neighboring city—that is, there is a positive spatial correlation between the urban carbon intensity of Chinese cities. (2) The direct effect of capital-saving technical change is greater than the indirect effect, and both the direct and indirect effects are significantly negative—that is, capital-saving technical change has a significant inhibitory effect on local and adjacent carbon intensity. (3) The capital-saving technical change of low-carbon cities has stronger inhibition on local carbon intensity than that of non-low-carbon cities.

Therefore, the policy implications of the results in this paper are as follows: First of all, China is now in a critical period of economic transformation and green development, which requires not only emphasizing and promoting the role of technical change in the coordinated development of urban economy, society, and ecology, but also adjustment of the direction of technical change in a timely manner, in order to contribute to the sustainable development of urban ecological environment in China. Secondly, we should focus on strengthening the interaction and linkage between cities, so that cities with low carbon intensity can play a leading and exemplary role, reducing the growth rate of carbon intensity in adjacent areas. Finally, it should be strongly advocated that the government and relevant departments should appropriately expand the scope of green policies, so that more Chinese cities benefit from the support of green policies and financial investment, and better develop urban energy conservation and emissions reduction activities.

## Figures and Tables

**Table 1:** Data description and source.

Variable	Name	Symbol	Data Source
Explained variables	Carbon intensity	*lncd*	The ratio of urban carbon emissions to GDP; the basic data can be found in *the China Urban Statistical Yearbook* (2009–2020) and *China Urban Environment Yearbook* (2009–2020).
Core explanatory variables	Directed technological change	*lndtc*	The capital stock is estimated by the perpetual inventory method based on the year 2000; total employment and real GDP are derived from the *China Urban Statistical Yearbook* (2009–2020) and *China Science and Technology Yearbook* (2009–2020).
Control variables	Population density (ratio of urban population to urban area)	*lnpop*	Data from the *Statistical Yearbook of Chinese Cities* (2009–2020).
	Average urban night lights	*lnnl*	NPP-VIIRS NTL (2014–2019) and DMSP-OLS RNTL (2009–2013), and using the method of Chen et al. (2021) to calibrate inconsistent data sources around 2013 [52].
	Foreign direct investment	*lnfdi*	Data from the *Statistical Yearbook of Chinese Cities* (2009–2020)
	The proportion of tertiary industry to GDP	*lnthird*	
	Total road passenger transport	*lntrans*	
	The number of full-time college teachers	*lntechs*	
	The ratio of pollution control investment to GDP	*lnpollution*	

**Table 2:** Statistical description of main variables.

Variable	Symbol	Unit	Observations	Mean	Standard Deviation	Minimum	Maximum
Carbon intensity	*lncd*	Tons/RMB 10,000	3113	0.87	2.3901	0.49	4.23
Directed technological change	*lndtc*	Data have been normalized	3113	0.65	0.7907	0.32	0.98
Population density (ratio of urban population to urban area)	*lnpop*	Number of people/hm^2^	3113	2.16	4.0014	1.57	3.05
Average urban night lights	*lnnl*	Candela(cd)	3113	3.70	5.0668	2.11	6.94
Foreign direct investment	*lnfdi*	RMB 10,000	3113	2.60	1.4762	1.70	5.08
The proportion of tertiary industry to GDP	*lnthird*	%	3113	37.8%	0.1825	28.71%	76.30%
Total road passenger transport	*lntrans*	10,000 people	3113	3.59	2.2003	1.04	6.68
The number of full-time college teachers	*lntechs*	10,000 people	3113	1.61	3.2947	2.69	5.74
The ratio of pollution control investment to GDP	*lnpollution*	RMB 10,000	3113	3.98	3.0898	0.91	6.37

**Table 3:** OLS regression results.

Explanatory Variable	Coefficient	*t*-Value
*lndtc*	−0.0162 ***	(−2.83)
*lnpop*	−0.0024 **	(−2.30)
*lnnl*	0.0043 ***	(4.37)
*lnfdi*	−0.0624 ***	(−4.92)
*lnthird*	0.0003 ***	(5.34)
*lntrans*	0.0048 ***	(4.98)
*lntechs*	−0.0055	(−0.84)
*lnpollution*	−0.2046 *	(−1.86)

Note: the value of *t* is in parentheses, and ***, **, and * are significant at the levels of 1%, 5%, and 10%, respectively.

**Table 4:** LM testing results.

	Time and Space Are Not Fixed	Time and Space Are Fixed	Time Is Fixed	Space Is Fixed
LM test of spatial lag effect (LMlag)	41.47 *** (0.000)	52.48 *** (0.000)	51.28 ** (0.000)	47.59 *** (0.000)
Robust LM test of spatial lag effect (R-LMlag)	18.93 *** (0.000)	46.71 *** (0.000)	30.38 ** (0.000)	25.73 *** (0.000)
LM test of spatial error effect (LMerr)	30.65 *** (0.000)	9.63 *** (0.000)	38.03 ** (0.000)	17.92 *** (0.000)
Robust LM test for spatial error effects (R-LMerr)	1.61 (0.107)	2.30 ** (0.021)	1.91 * (0.057)	29.37 *** (0.000)

Note: the value of *t* is in parentheses, and ***, **, and * are significant at the levels of 1%, 5%, and 10%, respectively.

**Table 5:** Results of the SDM.

Variable	Coeffcient	Lag Coefficient
	*β*	*θ*
Spatial regression coefficient δ	0.1027 *** (4.91)	−
*lndtc*	−0.0398 *** (−4.50)	−0.0458 *** (−8.28)
*lnpop*	−0.0624 *** (−3.66)	0.0620 *** (3.53)
*lnnl*	0.0426 *** (4.37)	0.3019 *** (6.89)
*lnfdi*	−0.0109 ** (−2.46)	0.3813 *** (9.37)
*ln3rd*	0.0167 *** (5.08)	0.0299 ** (2.31)
*lntrans*	1.5103 *** (5.34)	0.0374 *** (2.46)
*lntechs*	−0.0070 (−0.79)	−0.0903 (−1.21)
*lnpollution*	−0.7210 *** (−4.98)	0.4892 *** (−4.50)

Note: the value of *t* is in parentheses, and ***, ** are significant at the levels of 1%, 5%, respectively.

**Table 6:** Direct and indirect effects of the SDM.

Variable	Direct Effect	Indirect Effect	Total Effect
*lndtc*	−0.0472 *** (−4.69)	−0.0259 ** (−2.08)	−0.0731 *** (−3.99)
*lnpop*	0.4843 (0.56)	−0.5946 * (1.85)	−0.1103 * (1.90)
*Lnnl*	0.0305 ** (2.11)	0.5858 *** (3.99)	0.6163 *** (5.28)
*Lnfdi*	−0.1671 * (−1.83)	−0.0053 ** (−2.55)	−0.1724 *** (−6.21)
*Ln3rd*	−0.0951 *** (−4.93)	−0.0054 ** (−2.38)	−0.1005 *** (−4.91)
*lntrans*	0.2376 ** (2.11)	0.0374 *** (2.46)	0.2750 *** (3.90)
*lntechs*	0.0447 (0.16)	0.0013 (0.70)	0.0460 (0.99)
*lnpollution*	−0.8181 * (−1.83)	0.3024 *** (5.13)	−0.5157 *** (4.02)
*rho*	0.2857 ** (2.51)		
R^2^	0.68		
likelihood ratio	1329.0971		

Note: the value of *t* is in parentheses, and ***, **, and * are significant at the levels of 1%, 5%, and 10%, respectively.

**Table 7:** Low-carbon cities and non-low-carbon cities.

	Low-Carbon Cities			Non-Low-Carbon Cities		
Variable	Direct Effect	Indirect Effect	Total Effect	Direct Effect	Indirect Effect	Total Effect
*lndtc*	−0.5346 *** (−2.72)	−0.2616 *** (−3.10)	−0.7962 ** (−2.29)	−0.0436 ** (−2.29)	−0.0074 ** (−2.10)	−0.0510 ** (−1.79)
*lnpop*	−0.3478 ** (−2.13)	−0.1791 * (−1.69)	−0.5269 * (−1.80)	0.0421 ** (2.41)	0.0191 * (1.67)	0.0612 * (1.69)
*lnnl*	0.1200 *** (3.62)	0.0132 ** (3.30)	0.1332 ** (2.06)	0.0142 * (1.74)	0.0124 * (1.81)	0.0266 ** (2.40)
*lnfdi*	−0.1236 ** (−2.53)	−0.0981 ** (−2.07)	−0.2217 ** (−2.03)	−0.0188 *** (−5.75)	−0.0020 *** (−3.96)	−0.0208 *** (−2.85)
*ln3rd*	−0.1028 ** (−2.19)	0.0145 * (1.82)	0.0883 * (1.81)	0.0033 ** (2.23)	0.0174 * (1.83)	0.0207 ** (1.74)
*lntrans*	0.0337 ** (2.06)	0.0294 * (1.83)	0.0631 * (1.72)	0.0013 ** (2.20)	0.0066 * (1.89)	0.0079 ** (1.96)
*lntechs*	0.1394 (1.61)	0.0110 (0.17)	0.1504 (0.12)	0.0411 (1.04)	0.0007 (0.87)	0.0418 (0.14)
*lnpollution*	−1.1082 *** (−5.09)	−0.0201 *** (−2.85)	−1.1283 *** (−6.20)	0.1313 ** (1.96)	−0.0507 ** (−2.12)	0.0806 ** (2.24)
Rho	0.1793 ** (2.23)			0.2667 ** (2.50)		
R^2^	0.76			0.69		
Likelihood ratio	1319.8709			783.9702		

Note: the value of *t* is in parentheses, and ***, **, and * are significant at the levels of 1%, 5%, and 10%, respectively.

**Table 8:** Robustness test.

	Replacement of Geographic Weights		Dynamic Durbin Model		Replacement of the Explanatory Variable	
Variable	Direct Effect	Indirect Effect	Direct Effect	Indirect Effect	Direct Effect	Indirect Effect
*lndtc*	−1.0453 *** (−7.21)	−0.0726 ** (−2.23)	−0.0203 ** (−2.14)	−0.0169 * (−2.75)	−0.0266 *** (−2.48)	−0.0116 ** (−2.48)
*lnpop*	0.0068 ** (2.42)	0.0205 * (1.76)	0.0014 * (1.95)	0.0167 ** (2.10)	0.0031 *** (2.83)	0.0086 *** (4.87)
*lnnl*	0.0042 *** (2.95)	0.0076 ** (2.07)	0.1236 ** (2.47)	0.0009 ** (2.15)	0.0051 *** (3.77)	0.0312 *** (3.18)
*lnfdi*	−0.0136 ** (−2.26)	−0.0199 ** (−2.30)	−0.0410 *** (−4.39)	−0.0209 *** (−6.31)	−0.5134 ** (−2.39)	−0.7758 *** (5.19)
*ln3rd*	0.0019 ** (1.70)	0.0025 * (1.92)	0.0424 ** (2.07)	0.0086 *** (2.66)	0.0072 ** (2.23)	0.0058 ** (1.71)
*lntrans*	0.0414 *** (3.04)	0.0015 * (1.95)	0.0009 * (1.78)	0.0180 ** (2.05)	0.0027 * (1.73)	−0.0222 ** (1.88)
*lntechs*	0.0085 (0.94)	0.0106 (0.38)	0.0007 (0.96)	0.0281 (1.06)	0.0016 (0.55)	0.0459 (1.35)
*lnpollution*	−0.0709 *** (−4.39)	0.0172 *** (2.88)	−0.9781 *** (−4.22)	0.0338 ** (2.12)	−0.0404 ** (−2.41)	0.0016 ** (2.40)
Rho	0.2913 *** (2.80)		0.1433 *** (6.28)		0.0136 *** (8.50)	
R^2^	0.64		0.49		0.72	
Likelihood ratio	838.7348		1092.5382		1205.9276	

Note: the value of *t* is in parentheses, and ***, **, and * are significant at the levels of 1%, 5%, and 10%, respectively.

## Data Availability

Due to the confidentiality and privacy of the data, they will only be provided upon reasonable request.

## References

[1] Zemp M., Huss M., Thibert E., Eckert N., McNabb R.W., Huber J., Barandun M., Machguth H., Nussbaumer S.U., Gärtner-Roer I. (2019). Global glacier mass changes and their contributions to sea-level rise from 1961 to 2016. Nature.

[2] Stainforth D.A., Calel R. (2020). New priorities for climate science and climate economics in the 2020s. Nat. Commun..

[3] Li Z.-Z., Li R.Y.M., Malik M.Y., Murshed M., Khan Z., Umar M. (2021). Determinants of carbon emission in China: How good is green investment?. Sustain. Prod. Consum..

[4] Wang Y., Gong X. (2020). Does financial development have a non-linear impact on energy consumption? Evidence from 30 provinces in China. Energy Econ..

[5] He Y., Xu Y., Pang Y., Tian H., Wu R. (2016). A regulatory policy to promote renewable energy consumption in China: Review and future evolutionary path. Renew. Energy.

[6] Yu J., Shi X., Guo D., Yang L. (2021). Economic policy uncertainty (EPU) and firm carbon emissions: Evidence using a China provincial EPU index. Energy Econ..

[7] Chinowsky P., Hayles C., Schweikert A., Strzepek N., Strzepek K., Schlosser C.A. (2011). Climate change: Comparative impact on developing and developed countries. Eng. Proj. Organ. J..

[8] Porter M.E. (1990). Competitive Advantage of Nations? The Competitive Advantage of Nations.

[9] Sisco M.R., Pianta S., Weber E.U., Bosetti V. (2021). Global climate marches sharply raise attention to climate change: Analysis of climate search behavior in 46 countries. J. Environ. Psychol..

[10] Antonelli C., Feder C. (2020). The new direction of technological change in the global economy. Struct. Chang. Econ. Dyn..

[11] Nguyen T.T., Pham T.A.T., Tram H.T.X. (2020). Role of information and communication technologies and innovation in driving carbon emissions and economic growth in selected G-20 countries. J. Environ. Manag..

[12] Sajid M.J., Niu H., Liang Z., Xie J., Rahman M.H.U. (2021). Final consumer embedded carbon emissions and externalities: A case of Chinese consumers. Environ. Dev..

[13] Wang C., Zhang L., Zhou P., Chang Y., Zhou D., Pang M., Yin H. (2019). Assessing the environmental externalities for biomass- and coal-fired electricity generation in China: A supply chain perspective. J. Environ. Manag..

[14] Ke H., Dai S., Yu H. (2021). Spatial effect of innovation efficiency on ecological footprint: City-level empirical evidence from China. Environ. Technol. Innov..

[15] Khanna N., Fridley D., Hong L.X. (2014). China’s pilot low-carbon city initiative: A comparative assessment of national goals and local plans. Sustain. Cities Soc..

[16] Mimura T., Simayoshi H., Suda T., Iijima M., Mituoka S. (1997). Development of energy saving technology for flue gas carbon dioxide recovery in power plant by chemical absorption method and steam system. Energy Convers. Manag..

[17] Adhvaryu A., Kala N., Nyshadham A. (2020). The light and the heat: Productivity co-benefits of energy-saving technology. Rev. Econ. Stat..

[18] Almeida S.T.D., Borsato M. (2019). Assessing the efficiency of End of Life technology in waste treatment—A bibliometric literature review. Res. Conserv. Recycl..

[19] Iris Ç., Lam J.S.L. (2019). A review of energy efficiency in ports: Operational strategies, technologies and energy management systems. Renew. Sustain. Energy Rev..

[20] Shove E. (2018). What is wrong with energy efficiency?. Build. Res. Inf..

[21] Raza A., Gholami R., Rezaee R., Rasouli V., Rabiei M. (2019). Significant aspects of carbon capture and storage—A review. Petroleum.

[22] Singh A., Mishra N., Ali S.I., Shukla N., Shankar R. (2015). Cloud computing technology: Reducing carbon footprint in beef supply chain. Int. J. Prod. Econ..

[23] Hicks J.R.S. (1932). The Theory of Wages.

[24] Kennedy C. (1964). Induced bias in innovation and the theory of distribution. Econ. J..

[25] Samuelson P.A. (1965). Proof that properly anticipated prices fluctuate randomly. Ind. Manag. Rev..

[26] Drandakis E.M., Phelps E.S. (1966). A model of induced invention, growth and distribution. Econ. J..

[27] Acemoglu D. (1998). Why do new technologies complement skills? Directed technical change and wage inequality. Q. J. Econ..

[28] Acemoglu D. (2003). Labor and capital: Augmenting technical change. J. Eur. Econ. Assoc..

[29] Acemoglu D., Aghion P., Zilibotti F. (2006). Distance to frontier, selection, and economic growth. J. Eur. Econ. Assoc..

[30] Acemoglu D. (2007). Equilibrium bias of technology. Econometrica.

[31] Jones C.I. (2005). The shape of production function and the direction of technical change. Q. J. Econ..

[32] Acemoglu D. (2002). Directed technical change. Rev. Econ. Stud..

[33] Chen Y.H., Emer J., Sze V. (2016). Eyeriss: A spatial architecture for energy-efficient dataflow for convolutional neural networks. IEEE Micro..

[34] Huang J., Xiang S., Wu P., Chen X. (2022). How to control China’s energy consumption through technological progress: A spatial heterogeneous investigation. Energy.

[35] Yang Y., Liang S., Yang Y., Xie G.H., Zhao W. (2021). Spatial disparity of life-cycle greenhouse gas emissions from corn straw-based bioenergy production in China. Appl. Energy.

[36] Vega S.H., van Leeuwen E., van Twillert N. (2021). Uptake of residential energy efficiency measures and renewable energy: Do spatial factors matter?. Energy Policy.

[37] Yang L., Wang K.-L., Geng J.-C. (2018). China’s regional ecological energy efficiency and energy saving and pollution abatement potentials: An empirical analysis using epsilon-based measure model. J. Clean. Prod..

[38] Sun X. (2010). Research on fluctuations and convergency of efficiency in energy conservation and emission reduction in China. Stat. Inf. Forum.

[39] Yao X.L., Yu B. (2012). Technical progress, structure change, and carbon dioxide emissions of industry. Sci. Res. Manag..

[40] Wei W.X., Yang F. (2010). Impact of technology advance on carbon dioxide emission in China. Stat. Res..

[41] Färe R., Grosskopf S., Lovell C.A.K., Grifell-Tatjé E. (1997). Biased technical change and the malmquist productivity index. Scand. J. Econ..

[42] Domazlicky W. (1999). Total factor productivity growth in manufacturing: A regional approach using linear programming. Reg. Sci. Urban Econ..

[43] Pastor J.T., Lovell C.K., Aparicio J. (2019). Defining a new graph inefficiency measure for the proportional directional distance function and introducing a new Malmquist productivity index. Eur. J. Oper. Res..

[44] Kone A.C., Buke T. (2019). Factor analysis of projected carbon dioxide emissions according to the ipcc based sustainable emission scenario in turkey. Renew. Energy.

[45] Mulligan C., Sala-I-Martin X. (1995). A Labor-Income-Based Measure of the Value of Human Capital: An Application to the States of the United States.

[46] Lu F., Liu M.H., Sun Y.Y. (2018). Agglomeration, TFP and industrial growth. Stud. Sci. Sci..

[47] Wang B.B., Qi S.Z. (2014). Biased technological progress, factor substitution and China’s industrial energy intensity. Econ. Res. J..

[48] Tu Z.G., Chen L. (2019). The direction of technological progress and high-quality economic development: Based on the perspective of TFP and industrial structure upgrading. J. China Univ. Geosci..

[49] Liang T., Wang S., Lu C., Jiang N., Long W., Zhang M., Zhang R. (2020). Environmental impact evaluation of an iron and steel plant in China: Normalized data and direct/indirect contribution. J. Clean. Prod..

[50] Silva R., Lbo R., Faro L.E., Santos G., Peixoto M. (2020). Genetic parameters for somatic cell count (scc) and milk production traits of guzerá cows using data normalized by different procedures. Trop. Anim. Health Prod..

[51] Dietz T., Rosa E.A. (1994). Rethinking the environmental impacts of population, affluence and technology. Hum. Ecol. Rev..

[52] Chen J.D., Wang B., Huang S., Song M. (2020). The influence of increased population density in China on air pollution. Sci. Total Environ..

[53] Acemoglu D., Aghion P., Bursztyn L., Hemous D. (2012). The environment and directed technical change. Am. Econ. Rev..

[54] Stengos T., Fatouros N. (2020). Nuclear Energy, Economic Growth and the Environment: Optimal Policies in a Model with Endogenous Technical Change and Environmental Constraints.

[55] Wang X., Wang Y., Lan Y. (2021). Measuring the bias of technical change of industrial energy and environment productivity in China: A global DEA-Malmquist productivity approach. Environ. Sci. Pollut. Res..

[56] Bao C., Chen X.J., Liang G.L. (2016). Analysis on the influencing factors of water use efficiency in Henan province based on spatial econometric models. J. Nat. Resour..

[57] Anselin L., Le G.J., Jayet H., Matyas L., Sevestre P. (2006). Spatial panel econometrics. The Econometrics of Panel Data, Fundamentals and Recent Developments in Theory and Practice.

[58] Druska V., Horrace W.C. (2004). Generalized moments estimation for spatial panel data: Indonesian rice farming. Am. J. Agric. Econ..

[59] Baltagi B., Song S.H., Koh W. (2003). Testing panel data regression models with spatial error correlation. J. Econ..

[60] Anselin L., Hudak S. (1992). Spatial econometrics in practice: A review of software options. Reg. Sci. Urban Econ..

[61] Yang Y., Zhao T., Wang Y., Shi Z. (2015). Research on impacts of population-related factors on carbon emissions in Beijing from 1984 to 2012. Environ. Impact Assess. Rev..

[62] Bai C.Q., Zhou L., Xia M.L., Feng C. (2020). Analysis of the spatial association network structure of China’s transportation carbon emissions and its driving factors. J. Environ. Manag..

[63] Elhorst J.P. (2014). Matlab software for spatial panels. Int. Reg. Sci. Rev..

[64] Lesage J.P., Pace R.K. (2009). Introduction to Spatial Econometrics.

[65] Gómez-Rubio V., Bivand R.S., Rue H. (2021). Estimating spatial econometrics models with integrated nested laplace approximation. Mathematics.

[66] Anselin L., Bera A.K., Florax R., Yoon M.J. (1996). Simple diagnostic tests for spatial dependence. Reg. Sci. Urban Econ..

[67] Baltagi B.H., Li Q. (1995). Testing AR(1) against MA(1) disturbances in an error component model. J. Econ..

[68] Anselin L. (1988). Spatial Econometrics: Methods and Models.

[69] Zhang J.F., Fang Y. (2011). A robust test for spatial errors models. J. Quant. Tech. Econ..

